# Characterization of the murine macrophage response to infection with virulent and avirulent *Burkholderia* species

**DOI:** 10.1186/s12866-015-0593-3

**Published:** 2015-11-06

**Authors:** Chih-Yuan Chiang, Ricky L. Ulrich, Melanie P. Ulrich, Brett Eaton, Jenifer F. Ojeda, Douglas J. Lane, Krishna P. Kota, Tara A. Kenny, Jason T. Ladner, Samuel P. Dickson, Kathleen Kuehl, Rahul Raychaudhuri, Mei Sun, Sina Bavari, Mark J. Wolcott, David Covell, Rekha G. Panchal

**Affiliations:** Molecular and Translational Sciences Division, United States Army Medical Research Institute of Infectious Diseases (USAMRIID), Fort Detrick, MD USA; Institute for Therapeutic Innovation, Department of Medicine, University of Florida, Orlando, FL USA; Florida SouthWestern State College, Fort Myers, FL USA; Perkin Elmer, Waltham, MA USA; Center for Genome Sciences, USAMRIID, Fort Detrick, MD USA; Office of Regulated Studies, USAMRIID, Fort Detrick, MD USA; Pathology Division, USAMRIID, Fort Detrick, MD USA; Diagnostic Systems Division, USAMRIID, Fort Detrick, MD USA; Screening Technologies Branch, Developmental Therapeutics Program, National Cancer Institute, Frederick, MD USA

**Keywords:** *Burkholderia thailandensis*, *Burkholderia mallei*, *Burkholderia pseudomallei*, *Burkholderia oklahomensis*, host response

## Abstract

**Background:**

*Burkholderia pseudomallei* (Bp) and *Burkholderia mallei* (Bm) are Gram-negative facultative intracellular pathogens, which are the causative agents of melioidosis and glanders, respectively. Depending on the route of exposure, aerosol or transcutaneous, infection by Bp or Bm can result in an extensive range of disease – from acute to chronic, relapsing illness to fatal septicemia. Both diseases are associated with difficult diagnosis and high fatality rates. About ninety five percent of patients succumb to untreated septicemic infections and the fatality rate is 50 % even when standard antibiotic treatments are administered.

**Results:**

The goal of this study is to profile murine macrophage-mediated phenotypic and molecular responses that are characteristic to a collection of Bp, Bm, *Burkholderia thailandensis* (Bt) and *Burkholderia oklahomensis* (Bo) strains obtained from humans, animals, environment and geographically diverse locations. *Burkholderia* spp. (*N* = 21) were able to invade and replicate in macrophages, albeit to varying degrees. All Bp (*N* = 9) and four Bm strains were able to induce actin polymerization on the bacterial surface following infection. Several Bp and Bm strains showed reduced ability to induce multinucleated giant cell (MNGC) formation, while Bo and Bp 776 were unable to induce this phenotype. Measurement of host cytokine responses revealed a statistically significant Bm mediated IL-6 and IL-10 production compared to Bp strains. Hierarchical clustering of transcriptional data from 84 mouse cytokines, chemokines and their corresponding receptors identified 29 host genes as indicators of differential responses between the *Burkholderia* spp. Further validation confirmed Bm mediated *Il-1b*, *Il-10, Tnfrsf1b* and *Il-36a* mRNA expressions were significantly higher when compared to Bp and Bt.

**Conclusions:**

These results characterize the phenotypic and immunological differences in the host innate response to pathogenic and avirulent *Burkholderia* strains and provide insight into the phenotypic alterations and molecular targets underlying host-*Burkholderia* interactions.

**Electronic supplementary material:**

The online version of this article (doi:10.1186/s12866-015-0593-3) contains supplementary material, which is available to authorized users.

## Background

*Burkholderia pseudomallei* (Bp) and *Burkholderia mallei* (Bm) are Gram-negative facultative intracellular pathogens that cause melioidosis and glanders, respectively [1]. Bp causes disease in both humans and animals in the endemic areas of Southeastern Asia and Northern Australia [2, 3]. Infection with Bp can occur via percutaneous inoculation, injection or inhalation of aerosolized bacteria or contaminated fluids [1]. Clinical signs of melioidosis may manifest as flu-like symptoms, pneumonia, or fulminating septicemia that are often fatal [1, 4]. Both chronic and acute forms of melioidosis have been reported and the pathogen can colonize a diverse range of tissues including liver, spleen, lung, skin and even the urinary tract.

Bm is a non-motile, obligate mammalian pathogen that is closely related at the genetic level to the much more diverse species Bp [1, 5, 6]. The pathogen is endemic among domestic animals in Africa, Asia, the Middle East and Central and South America [6]. Horses are the natural reservoir for Bm, but mules and donkeys are also susceptible [7]. Equine infections are caused by consumption of water or feed contaminated with nasal discharge from infected equines, but a cutaneous form of the disease, known as farcy, also exists. Human infection is primarily caused by direct contact with an infected animal’s nasal discharge or skin lesion exudates [8]. In humans, glanders is characterized initially by the onset of fever, rigors and malaise, rapidly leading to pneumonia, bacteremia, pustules and abscesses. Due to the highly infectious nature of Bp and Bm, in particular for exposure by the aerosol route, both pathogens are considered potential biological warfare threat agents and are classified by the federal select agent program as Tier 1 select agents.

Two other Bp-like species that are closely related, at the genetic and physiological level, that have been reported are Bo and Bt [9, 10]. Bo was first isolated in Oklahoma in 1973 from the purulent discharge of a pelvic wound from a farmer that was involved in a tractor accident [9, 11]. Initially, Bo was described as Bp due to the similar metabolic and culture conditions but was differentiated from Bp using serology and fatty acid composition analysis. Bo is avirulent in hamster and mouse models [12]. Bt CDC2721121 and Bt CDC3015869 were isolated from patient samples in Louisiana and Texas, respectively. Although both Bt CDC2721121 and Bt CDC3015869 originated from human source in the United States of America, they displayed different in vivo pathogenicity profiles. It was demonstrated in Syrian hamster model that Bt CDC2721121 was avirulent whereas Bt CDC3015869 had a virulence capacity that was very similar to the Bt Phuket 4 W-1 strain [12]. Numerous differences between Bp and Bt were reported at the genetic, phenotypic, and pathogenic level [12–14]. However, host responses to these pathogens have not been well characterized.

A characteristic feature of Bp, Bm, Bt and Bo pathogens is their ability to infect both phagocytic and nonphagocytic host cells [15, 16]. The intracellular life cycle of these pathogens involves a coordinated interaction between the host and pathogen proteins that allows the bacteria to adhere and gain entry into the phagosomal compartment of the target cells [17]. Disruption of the phagosomal membrane by Bsa type III secretion system (T3SS) allows the bacteria to escape into the host cytosol, evade host innate responses and killing by autophagy. In the host cytoplasm, bacteria gain motility and spread from cell-to-cell via the polymerization of host actin; a process directed by the bacterial cell surface protein, BimA [18–22]. In contrast, Bt can employ a cryptic (fla2) flagellar system and drive cell-to-cell spread in a BimA independent manner [23]. The type VI secretion system (T6SS) also plays a critical role in bacterial replication and intercellular spread by inducing fusion of the plasma membrane of the infected host cells to form multinucleated giant cells (MNGCs), a hallmark of *Burkholderia* infection which has been observed in phagocytic, nonphagocytic cell lines and clinical glanders and melioidosis samples. [1, 19]. The expression of the T3SS and T6SS is controlled by mechanisms such as TetR-type regulator, two component systems or quorum sensing [24–26]. Host pathogen recognition receptors (PRRs) and their associated molecules such as Toll-like receptors (TLRs), NOD-like receptors (NLRs) and caspases play a pivotal role in *Burkholderia* spp. infection [27–34]. Likewise, MCP-1, interferon (IFN)-γ, TNF-α, IL-6 and IL-10 are key cytokines that modulate Bp and Bm infection [28, 35–38]. Host innate immune signaling cascades, however, can be counteracted by bacterial virulence factors. For example, Bp encoded TssM downregulates host inflammatory responses by inhibiting NF-κB and Type I IFN pathway activation [39], while BopA is important for avoidance of autophagy [26]. Similarly, Cif homolog in Bp (CHBP) also abrogates NF-κB activation by deregulating IκBα degradation and p65 nuclear translocation [40]. Although the interactions between *Burkholderia* spp. and its hosts have been examined previously, host innate immune responses that are associated with individual strains of *Burkholderia* spp. is lacking.

The goal of this study was to conduct systematic analyses of the host phenotypic alterations at the cellular level and immunological responses at the molecular level using a diverse collection of *Burkholderia* spp. obtained from humans, animals, environment and geographically diverse locations. Several strains used in this study have been previously characterized for their pathogenicity in vivo in mice or in the Syrian hamster model of infection [12, 41–43]. Measurement of phenotypic responses using conventional colony forming unit (CFU) assays and high-content imaging (HCI) assay demonstrated all *Burkholderia* spp. were phagocytosed and replicated within RAW264.7 macrophages. Bo E0147 and Bp 776 failed to induce MNGCs whereas all other *Burkholderia* spp. induced MNGC to varying degrees. Elevated production of IL-1β, TNF-α and KC (murine homolog of human IL-8) cytokines was observed in all *Burkholderia* spp. infected macrophages. On the contrary, the secretion of IL-6 and IL-10 was significantly higher in Bm infected macrophages than that of Bp. Hierarchical clustering of the gene expression data from 84 inflammation related genes revealed 29 genes as indicators of differential responses between *Burkholderia* strains. Further validation studies confirmed a significantly elevated Bm mediated *Il-1b*, *Il-10, Tnfrsf1b* and *Il-36a* mRNA expressions compared to that with Bp and Bt. Collectively, these multidisciplinary approaches provided a comprehensive assessment of the murine macrophage host response(s) during different stages of infection with a diverse collection of *Burkholderia* spp.

## Results

### Diverse *Burkholderia* spp. are phagocytized and replicate within RAW264.7 macrophages

**Fig. 1:**
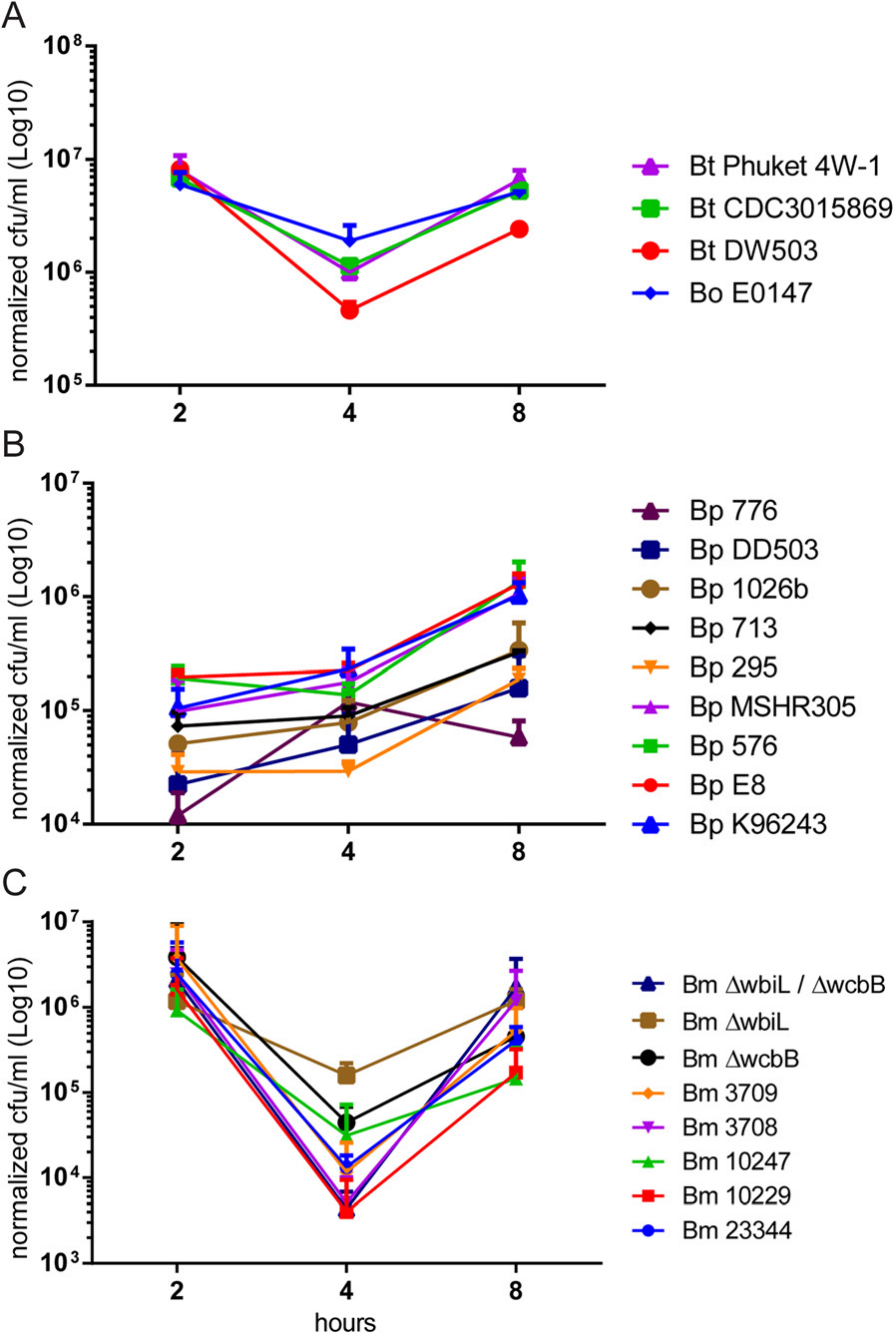
Quantitation of *Burkholderia* spp. intracellular replication. RAW264.7 macrophages were infected with the indicated Burkholderia spp. at a MOI of 10. Two hours post infection, kanamycin was added to reduce the growth of extracellular bacteria. Viable bacteria **a**) Bt and Bo; **b**) Bp; **c**) Bm were quantified at the indicated time points using CFU assays. All data shown is representative of two replicates per strain per time point, and performed on two independent days. Data was normalized and averaged by actual MOI

A diverse collection of Bp (N = 9), Bm (N = 5), Bt (N = 3) and Bo (N = 1) strains from various geographical locations throughout the world were examined in this study. Available information on the ancestry of the strains along with their source, location, in vivo virulence profile and genome sequences is listed in Table 1. In addition, three mutants of Bm ATCC 23344 strains with deletions in the genes encoding for capsule (Bm ATCC 23344 Δ*wcbB*) or lipopolysaccharide (LPS) (Bm ATCC 23344 Δ*wbiL*) biosynthesis or both (Bm ATCC 23344 Δ*wcbB/*Δ*wbiL*) were also evaluated. The ability of each strain to replicate within murine macrophages was determined by incorporating well-established kanamycin (Km) protection assays [44–47]. Intracellular replication within RAW264.7 macrophages was monitored at 2, 4 and 8 h post infection for each *Burkholderia* strains. The three Bt strains CDC3015869, Phuket 4 W-1, DW503 and Bo E0147 were internalized and able to replicate in RAW264.7 macrophages (Fig. 1a). Among all the Bp strains that were tested, no statistically significant differences were observed in the uptake and intracellular replication. However, macrophages infected with Bp 776 showed a much-reduced uptake of these bacteria at 2 h post infection, followed by a robust replication at 4 h and subsequent drop at 8 h post infection (Fig. 1b). Based on Transmission electron microscopy (TEM) studies, a much reduced number of the Bp 776 was observed in the cytosol compared to the reference strain Bp K96243 at the late time points (6 and 8 h) post infection, while very similar number of bacteria were observed within the membrane bound vesicles for both the strains (Additional file 1: Figure S1). These studies suggest that differential escape rate from endosomal compartment may contribute to the observed phenotype. The uptake of all five Bm strains was similar at the two hour time point. However, at the 4 and 8 h time points, differences were observed in the intracellular survival and replication but were not statistically significant (Fig. 1c). Furthermore, the intracellular replication of the polar Bm LPS and capsule mutants was similar to that observed for Bm ATCC 23344 (Fig. 1c).

**Table 1:** Bacterial strains used in this investigation and macrophage phenotypes following infection

Species and strain	Source	Location	Year isolated	Genome sequenced	Actin tails	MNGC	Uptake	Intracellular replication	In vivo Pathogenicity
*B. thailandensis*									
^a^DW503	Environment	Thailand	1998	Yes	Yes [44, 66]	Yes [44]	Yes [44]	Yes [44]	
CDC3015869	Human blood	Texas	2003	Yes	Yes [44]	Yes [44]	Yes [44]	Yes [44]	Virulent [12]
Phuket 4 W-1	Water	Thailand	Unknown	Partial	Yes [44]	Yes [44]	Yes [44]	Yes [44]	Virulent [12]
*B. oklahomensis*									
E0147	Human conjunctiva	Georgia, US	1977	Yes	No [44]	No [44]	Yes [44]	Yes [44]	Avirulent [12]
*B. mallei*									
NCTC 10229	Unknown	Hungary	1961	Yes	Yes	Yes	Yes	Yes	Virulent [41]
NCTC 10247	Unknown	Turkey	1960	Yes	No	Reduced	Yes	Yes	Attenuated [41]
NCTC 3708	Mule	India	1932	No	Yes	Yes	Yes	Yes	Virulent [41]
NCTC 3709	Horse	India	1932	Yes	Yes	Reduced	Yes	Yes	Virulent [41]
ATCC 23344	Human	China	1942	Yes	Yes [67]	Yes [68]	Yes [68]	Yes	Virulent [41]
^b^23344 Δ*wcbB*	UGA	China	1942	Yes	^c^NT	Reduced	Yes	Reduced	
^b^23344 Δ*wbiL*	UGA	China	1942	Yes	^c^NT	Yes	Yes	Reduced	
^b^23344 Δ*wbiL*/Δ*wcbB*	UGA	China	1942	Yes	^c^NT	Reduced	Yes	Reduced	
*B. pseudomallei*									
576	Human Blood	Thailand	Unknown	Yes	Yes [44]	Reduced	Yes [44]	Yes [44]	
MSHR305	Human Brain	Australia	1994	Yes	Yes	Yes	Yes	Yes	Virulent [43]
295	Soil	Australia	Unknown	No	Yes	Reduced	Reduced	Reduced	
713	Ulcer	Australia	Unknown	No	Yes	Reduced	Yes	Yes	
1026b	Blood	Thailand	1993	Yes	Yes [69]	Reduced	Yes [69]	Reduced	Virulent [43]
DD503	Soil	Australia	Unknown	No	Yes	Reduced	Reduced	Reduced	
776	Blood	Australia	Unknown	No	Yes	No	Reduced	Reduced	
E8	Soil	Thailand	1990	Yes	Yes [21]	Yes	Yes [21]	Yes [21]	
K96243	Human	Thailand	1996	Yes	Yes [44]	Yes [48]	Yes [44]	Yes [44]	Virulent [42]

Table 1 Bacterial strains used in this investigation and RAW264.7 macrophage phenotypes following infection. Explanations of column headings are: Species and strain: the species and strain that are used in this study; Source, location and year isolated: the source, location, and year that the corresponding strain was first identified; Genome Sequenced: the availability of the gene sequencing information for the corresponding strain; Actin tails, the ability of the corresponding strain to polymerize host actin and exhibit actin tails on the bacterial surface; MNGC, ability of the corresponding strain to induce macrophage MNGC; uptake and intracellular replication: ability of the corresponding strain to be taken up by macrophages and replicate intracellularly; in vivo pathogenicity, pathogenicity is determined by survival of mice and Syrian hamsters challenged with indicated *Burkholderia* spp.

^a^Derived from *B. thailandensis* E264; Δ(*amrR*-*oprA*) (Km^s^ Gm^s^ Sm^s^); *rpsL* (Sm^r^) [10]

^b^Obtained from the laboratory of Mark Schell, University of Georgia nUGA) and derived from *B. mallei* ATCC 23344

^c^NT, not tested in the study

Given the diverse collection of *Burkholderia spp.* analyzed in this investigation for bacterial uptake and intracellular replication, no distinct patterns linking strains isolated from humans, animals, environment (soil or water) nor geographic location (i.e. Thailand vs. Australia) were observed.

### Ability to induce macrophage MNGC formation and exhibit actin tails varies between *Burkholderia* spp. examined

**Fig. 2:**
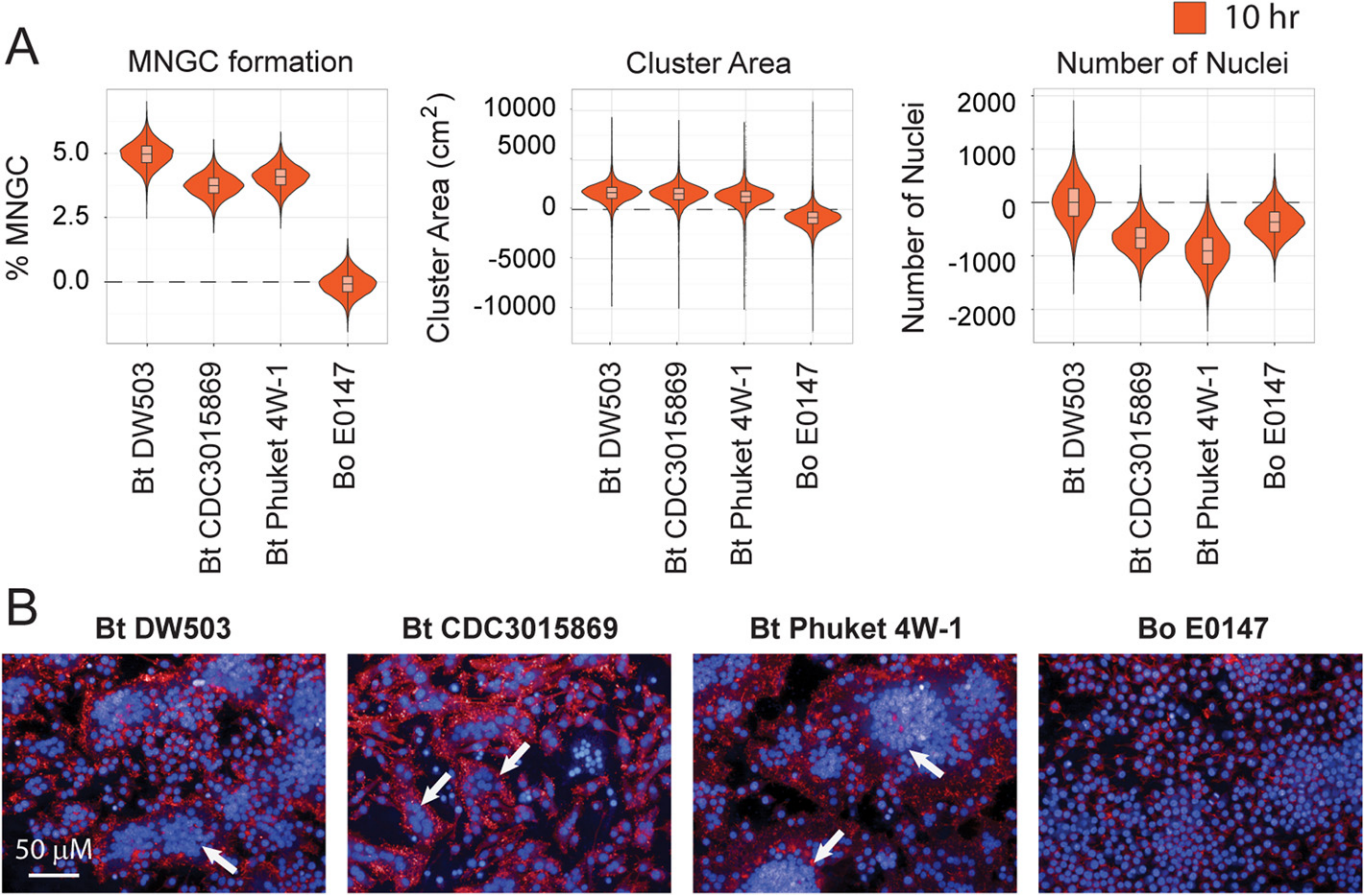
Quantitation of Bt and Bo induced MNGC formation. RAW264.7 macrophages were infected with indicated Bt and Bo strains at a MOI of 30. Ten hours post infection cells were fixed and stained with hoechst dye and phalloidin-568. **a**) Violin plots representing the quantitation of cellular attributes of the cluster population (i.e.: MNGC formation, Cluster Area and number of nuclei) as measured by MNGC image analyses procedure. All data was normalized to uninfected control. **b**) Representative confocal images of MNGC formation. Nuclei are pseudocolored blue (hoechst dye) and actin pseudocolored red (Phalloidin). Scale bar - 50 μm. All data shown is representative of six replicates per plate, three plates per day and performed on three independent days. White arrows indicate MNGCs

**Fig. 3:**
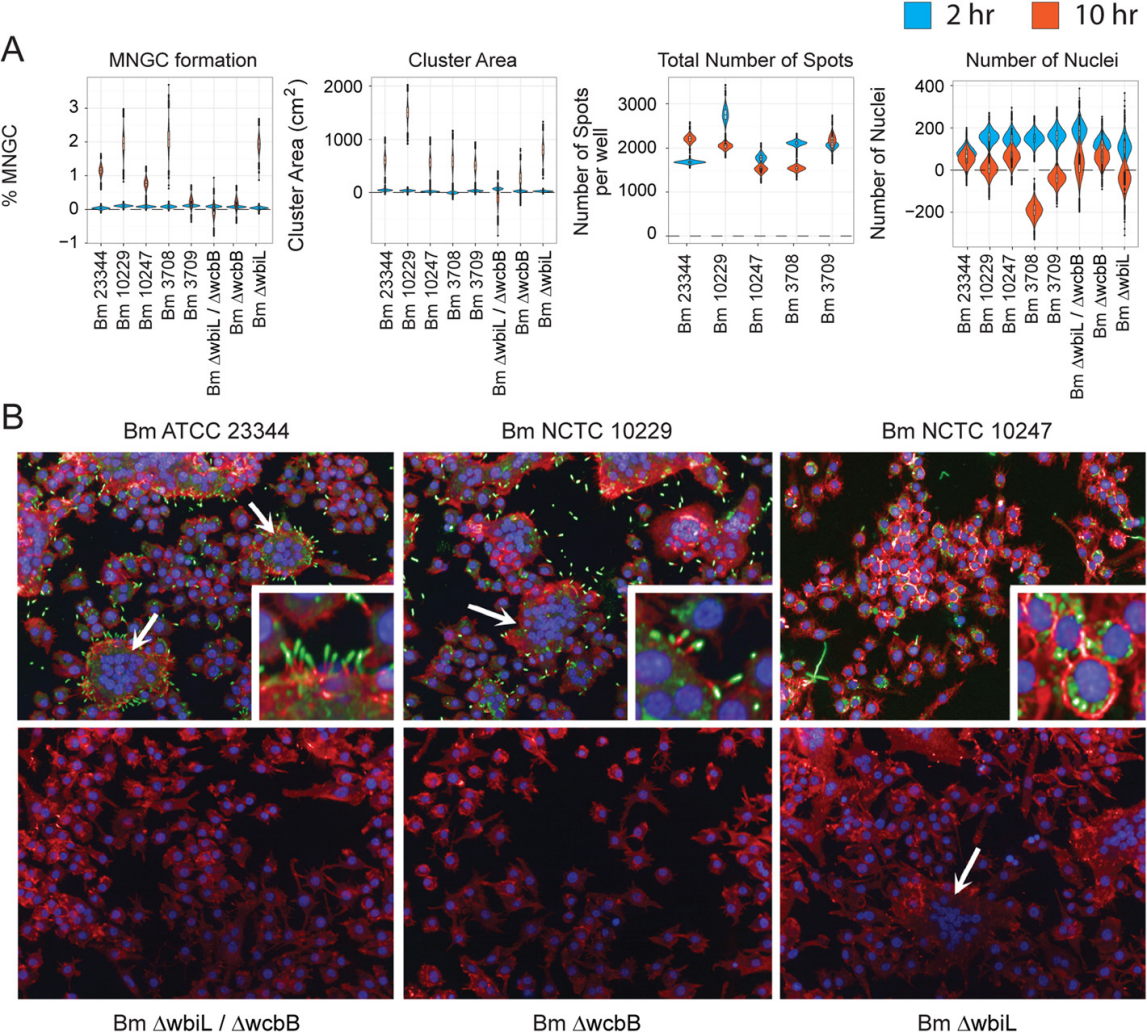
Quantitation of Bm induced MNGC formation. RAW264.7 macrophages were infected with indicated Bm strain at a MOI of 30. Two hours or ten hours post infection cells were fixed and stained with antibodies that detect the bacteria or the host actin tails on bacterial surface. Image acquisition and analysis were performed as described in Fig. 2. **a**) Violin plots representing the quantitation of cellular attributes of the cluster population as measured by MNGC image analyses procedure (See Fig. 2a). **b**) Representative confocal images of MNGC and actin tail formations. Nuclei are pseudocolored blue (hoechst dye), actin pseudocolored red (Phalloidin) and bacteria pseudocolored green (antibody). Scale bar - 50 μm. Data shown is representative of six replicates per plate, three plates per day and performed on three independent days. White arrows indicate MNGCs

**Fig. 4:**
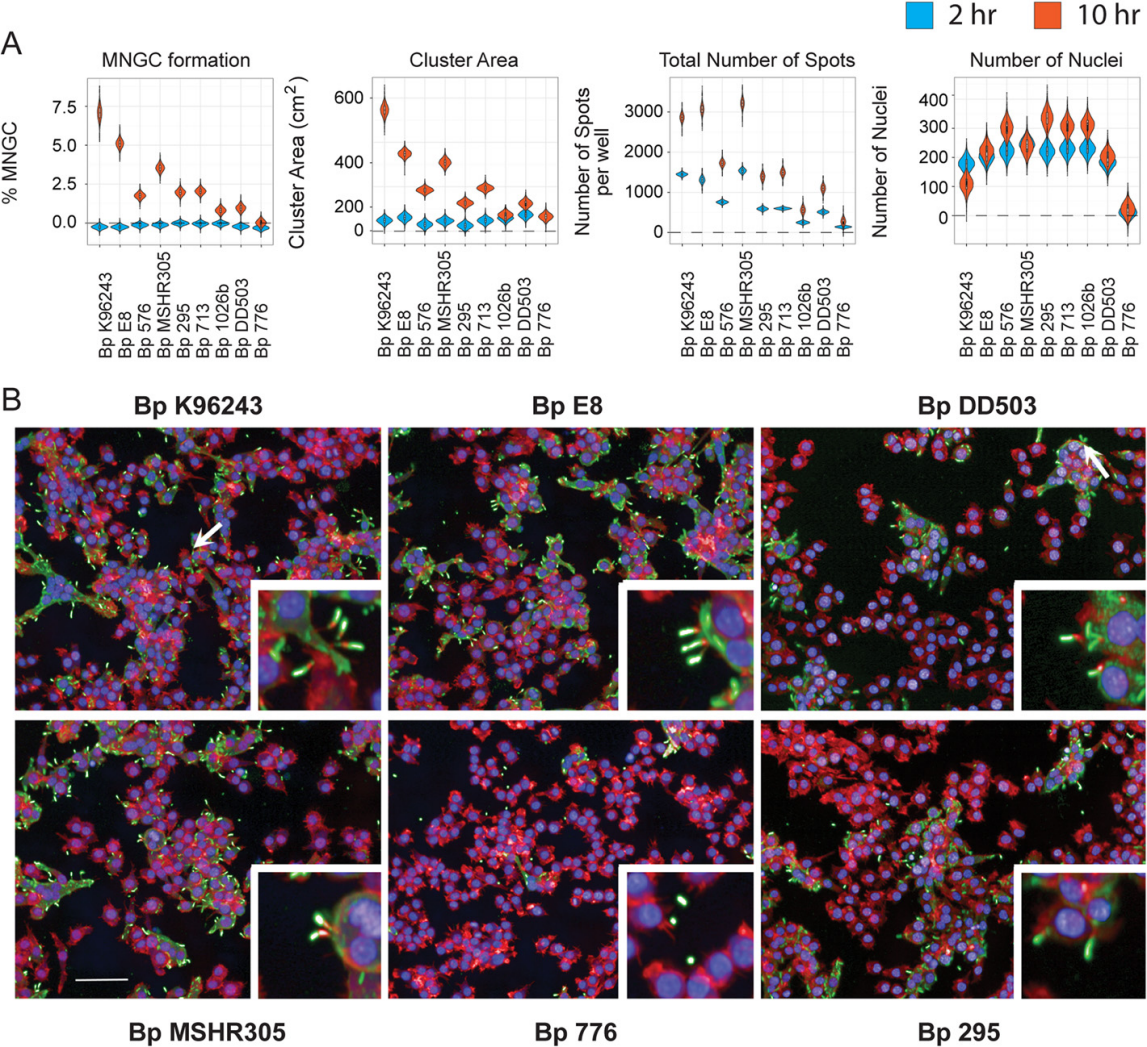
Quantitation of Bp induced MNGC formation. RAW264.7 macrophages were infected with indicated Bp strain at a MOI of 30. Two hours or ten hours post infection cells were fixed and stained with antibodies that detect the bacteria or the host actin tails on bacterial surface. Image acquisition and analysis were performed as described in Fig. 2. **a**) Violin plots representing the quantitation of cellular attributes of the cluster population as measured by MNGC image analyses procedure. **b**) Representative confocal images of MNGC and actin tail formations. Nuclei are pseudocolored blue (hoechst dye), actin pseudocolored red (Phalloidin) and bacteria pseudocolored green (antibody). Scale bar - 50 μm. Data shown is representative of six replicates per plate, three plates per day and performed on three independent days. White arrows indicate MNGCs

A hallmark of *Burkholderia* spp. infections is the ability of the bacteria to induce MNGC formation of infected macrophages, following cellular uptake and intracellular bacterial replication [17]. In this study, phenotypic screening using HCI was used to quantitate the MNGC phenotype [48]. All the three Bt strains were capable of inducing MNGCs in infected macrophages (Fig. 2a and b). The failure of Bo E0147 to cause MNGCs (Fig. 2a and b; Table 1; Additional file 2: Figure S2) was consistent with the phenotype reported by Wand et al. [44]. Among the five Bm strains, Bm NCTC 3709 and Bm NCTC 10247 exhibited much reduced ability to induce MNGC phenotype (Fig. 3a and b; Additional file 2: Figure S2). Interestingly, Bm 10247 bacteria appear to be trapped in the endocytic vesicles (Fig. 3b), a phenotype not observed following Bm 3709 infection (data not shown). The Bm capsule mutant Bm ATCC 23344 Δ*wcbB* and double deletion mutant Bm ATCC 23344 Δ*wcbB/*Δ*wbiL* also exhibited reduced ability to induce MNGC compared to the parental strain Bm ATCC 23344 (Fig. 3a and b; Additional file 2: Figure S2). Five out of the nine Bp strains exhibited reduced ability to induce MNGC when compared with Bp K96243 and Bp E8 (Fig. 4a and b, Table 1 and Additional file 2: Figure S2). The absence of MNGCs in Bp 776 infected RAW264.7 macrophages correlated with reduced uptake and intracellular replication as measured in the CFU assay and reduced endosomal escape rate by TEM.

Several bacterial pathogens, including *Shigella*, *Listeria*, *Mycobacteria* and *Burkholderia* induce host cell actin tail formation on the bacterial surface to facilitate cell-to-cell spread, while evading the host immunological responses and promoting intracellular replication [20]. The three Bt and four Bm strains as well as all the nine Bp strains (Figs. 2b, 3b and 4b and Table 1) tested in this study were capable of inducing host actin polymerization and exhibiting actin tails on the bacterial surface. However, Bo E0147 and Bm NCTC 10247 strains failed to exhibit actin tail formation on bacterial surface following infection (Table 1).

### *Burkholderia* spp. infected RAW264.7 macrophages induce differential cytokine responses

**Fig. 5:**
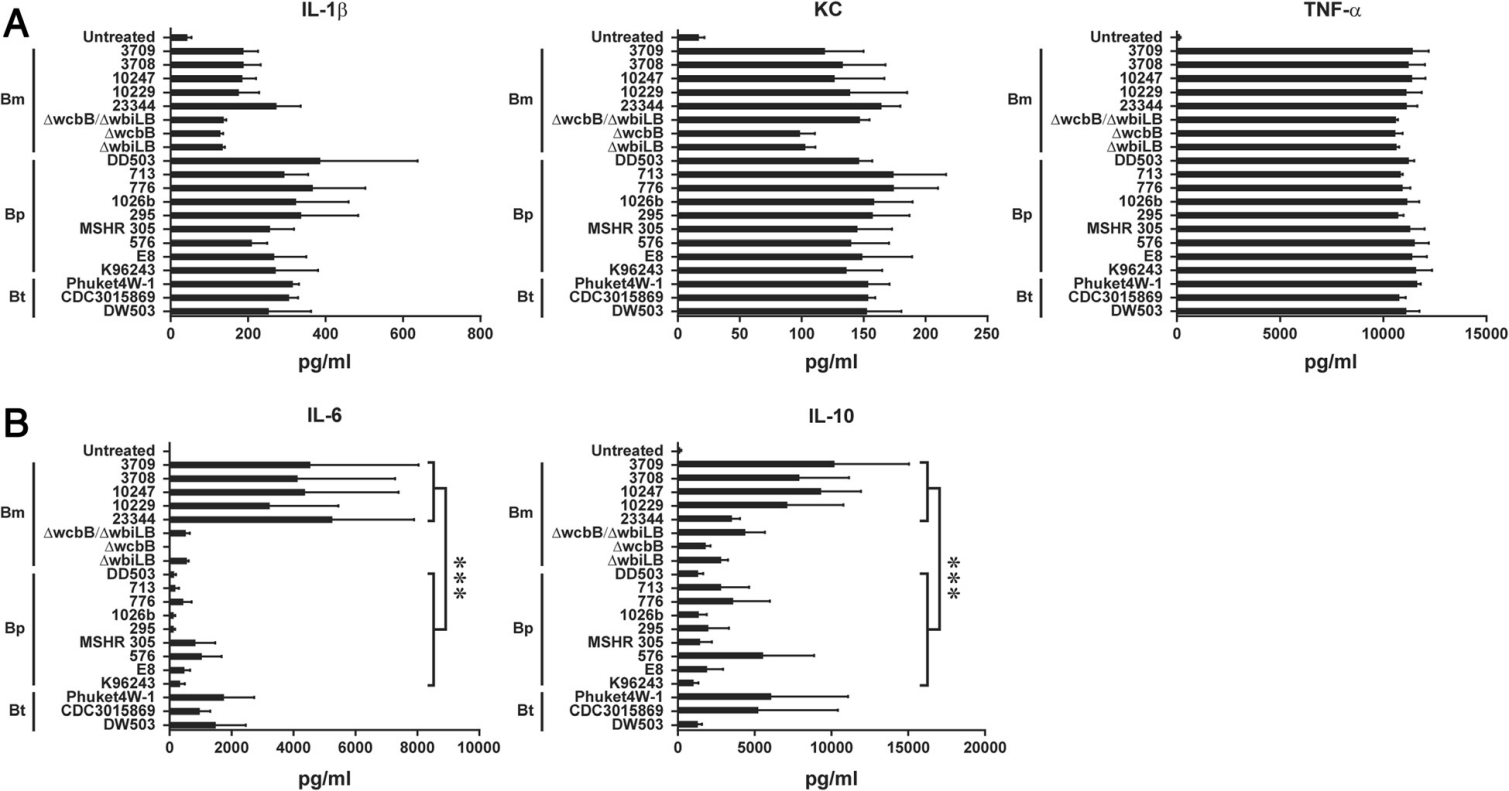
Profiling inflammation related cytokine production in RAW264.7 macrophages infected with *Burkholderia* spp. **a** & **b**) RAW264.7 macrophages were infected with listed *Burkholderia* spp. at a MOI of 10. Eight hours post infection, the supernatants were harvested and the amount of indicated cytokines was quantified. Unpaired Student’s *t*-test was performed to evaluate the statistical significance between the species (*** represent *p* value smaller than 0.001). All data shown is representative of n = 5 experiments

Previously, elevated IL-1β, IL-8, IL-6, TNF-α and IFN-γ concentrations have been associated with death among patients with melioidosis [49–51]. In addition, IL-10, a potent anti-inflammatory cytokine, was thought to have an important suppressive immune-regulatory role in the early stages of Bp infection [52]. The ability of *Burkholderia* spp. to modulate IL-1β, TNF-α, KC, IL-6 and IL-10 production in RAW264.7 macrophages was investigated. All twenty-one strains of *Burkholderia* spp. induced IL-1β, TNF-α and KC production (Fig. 5a and Additional file 3: Figure S3). Notably, RAW264.7 macrophages infected with Bm spp. resulted in significantly elevated levels of IL-6 and IL-10 when compared with Bp spp. infections (Fig. 5b). The production of IL-6 was reduced in RAW264.7 macrophages infected with mutant Bm strains (Bm ATCC 23344 Δ*wcbB*, Δ*wbiL*/Δ*wcbB* and Δ*wbiL*) compared to cells infected with the parental Bm ATCC 23344 strain. The observed difference in IL-6 and IL-10 production between Bm and Bp infected macrophages suggests the existence of distinct underlying molecular signaling cascades.

### Differential expression of inflammation related genes in *Burkholderia spp.* infected RAW264.7 macrophages

**Fig. 6:**
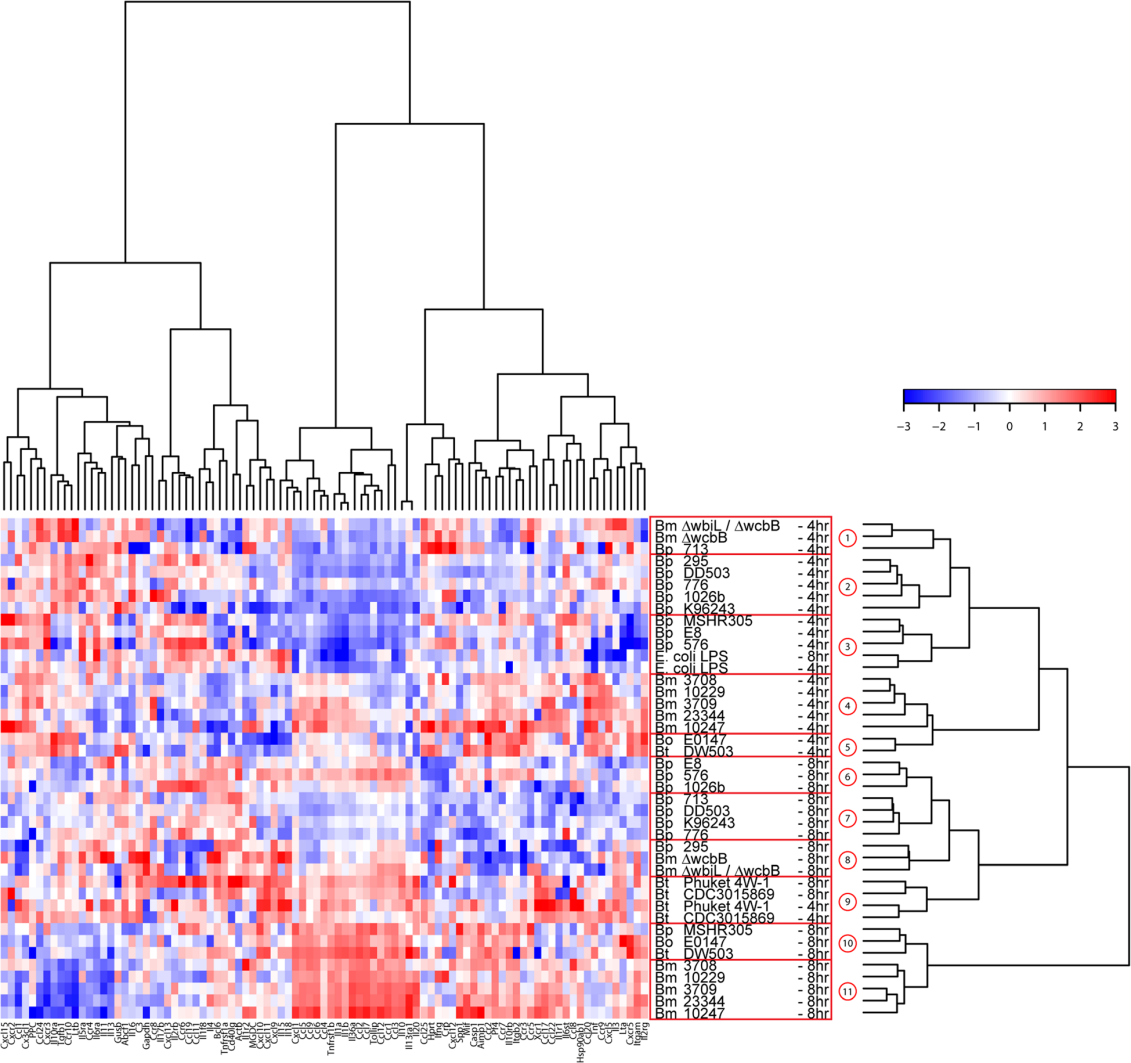
Differential expression of inflammation related genes following Bp, Bt and Bm infection. RAW264.7 macrophages were infected with indicated *Burkholderia* spp. at a MOI of 10. Total mRNA was purified and reverse transcribed to cDNA. A panel of 84 pro-inflammatory genes was quantified using real time PCR. Fold changes of gene expression are color coded, where red stands for high values and blue for low fold changes. Implementation of Manhattan distance calculation and a Wards linkage analysis identified eleven clades, which are highlighted in red box. The data is average of three independent experiments

**Fig. 7:**
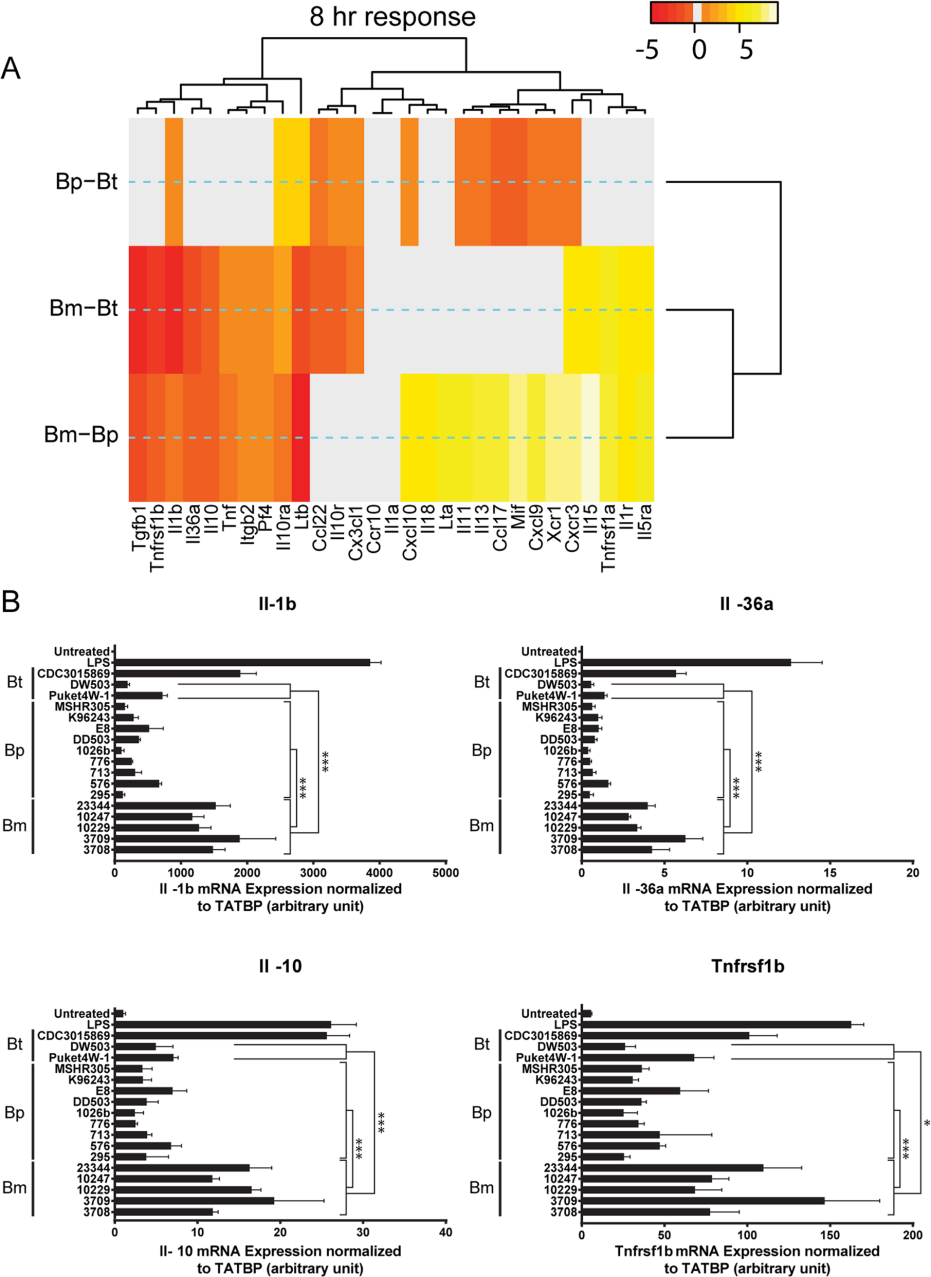
Heat map of pairwise differential gene expression and validation **a**) Heat maps are colored spectrally to indicate strength of statistical significance for pairwise comparisons. Spectral colors range from red (negative) to yellow (positive) for the t-statistic of each Student’s *t*-tests. Gray regions indicate absence of statistically significant differences in gene expression. Data for the 8 h time post infection is shown. **b**) mRNA was extracted from independently prepared RAW264.7 macrophages infected with *Burkholderia* spp. for eight hours. mRNA was reverse transcribed to cDNA and the expression levels of indicated genes were quantified by real-time PCR. (* and *** represent *p* value smaller than 0.05 and 0.001, respectively). The data is average of three independent experiments

Genes encoding chemokines and pro-inflammatory cytokines are critical mediators of intracellular bacterial infection for *in vivo* and *in vitro* systems. To determine if the different *Burkholderia* spp. modulate common or unique host immune responses, we examined the transcriptional profile of 84 inflammation related genes from RAW264.7 macrophages infected for 4 or 8 h with the diverse *Burkholderia* strains. Hierarchical clustering of the gene expression data separated *Burkholderia* strains into eleven sub-clades, with distinct segregation between the early (4 h and representing clades 1–5) and the late (8 h and representing clades 6–11) infection times (Fig. 6 and Additional file 4: Table S1). Sub-clustering within the early and late exposure times separated for the most part the Bm, Bp and Bt strains. Pairwise comparisons of host gene expressions at each exposure time were used to identify differences in the host responses to specific Bp, Bm and Bt species. A Student’s t statistic was computed and clustered heat map generated for the three pairwise gene expressions; Bp-Bt, Bm-Bt and Bm-Bp, using their corresponding pairwise differences between averaged gene expressions. Twenty-nine host genes were identified as indicators of differential responses between the *Burkholderia* spp. (Fig. 7a and Additional file 5: Table S2). Statistical analysis demonstrated that Bm collectively induced significantly higher expression of a gene cluster than Bp and Bt at 8 h post infection. The expression levels of a subset of genes, *Tnfrsf1b*, *Il-1b*, *Il-36a* and *Il-10* were validated by real-time PCR using independently prepared samples (Fig. 7b). With the exception of Bt CDC3015869, the Bm strains collectively showed statistically significant increased expression of *Tnfrsf1b*, *Il-1b*, *Il-36a* and *Il-10* genes compared to the Bp and the two Bt strains*.* These results indicate that gene-based differences for *Burkholderia* species are evident within the different components of the inflammatory response.

## Discussion

*In vitro* characterization of host responses to *Burkholderia* infection at both the phenotypic and molecular level provides a rapid approach to gain insight into the intracellular lifestyle of both pathogenic and avirulent *Burkholderia* strains. We characterized the host phenotypic responses by measuring each *Burkholderia* strain’s capacity to a) invade and replicate in macrophages, b) induce host actin polymerization on bacterial surfaces and c) induce RAW264.7 macrophage MNGC formation. All *Burkholderia* spp. invaded and replicated within RAW264.7 macrophages, albeit to varying degrees. Quantitation of MNGC formation in infected macrophages revealed several Bm (Bm NCTC 3709, Bm NCTC 10247) and Bp (Bp 576, Bp 295, Bp 713, Bp 1026b and Bp DD503) strains to exhibit a reduced capability of inducing this phenotype. There was no observed correlation between intracellular bacterial replication and reduced number of MNGCs or actin tails. *In vitro*, Bo E0147 strain is not capable of inducing MNGC phenotype, an observation similar to that reported by Wand et al. [44], and which in part could be due to its inability to polymerize host actin and thereby prevent the bacterial cell-to-cell spread. Prior published studies suggest that the lack of WASP homology 2 (WH2) domain in BimA and the absence of T3SS transcription regulators, BPSS1553 (bprP) and BPSS1554 (bprQ), in Bo may contribute to the observed phenotypes [44, 53]. Intriguingly, Bp 776 did not induce macrophage MNGC formation despite the detection of actin tail on the bacterial surface. Due to the lack of genome sequence information for Bp 776, the genome integrity cannot be determined and hence difficult to correlate phenotype with genotype. Bm NCTC 10247 did not exhibit actin tails, were trapped in endocytic compartments and showed much reduced capacity to induce MNGCs. Functional studies to correlate the Bm 10247 phenotype to genotype are ongoing.

Intrinsic differences at the cellular level may contribute to differences in host susceptibility to *Burkholderia* infection. For example, prior published studies have shown that C57BL/6 mice are 10 to 100 fold more resistant to Bp infection compared to BALB/c mice [52, 54, 55]. Furthermore, bone marrow-derived macrophages from C57BL/6 mice can clear the bacteria more efficiently compared to those obtained from BALB/c mice. In this study, since a uniformed *in vitro* system such as the RAW264.7 macrophages was used to characterize the host responses to *Burkholderia* spp. infection, differences in the bacterial genome composition or mutations may contribute to the observed cellular phenotypes. In an attempt to correlate the pathogen induced host phenotypes to mutations in known bacterial virulence factors, comparative genomics analysis was conducted for the Bm and Bp strains whose genome sequences were available. A little over 60 loci were examined in each species (Additional file 6: Table S3). These encompassed loci that were important for the expressions of T3SS-3, T6SS-1, actin motility, and several regulators of these systems including VirAG, BspR, BprP, BprQ, BsaN, and RpoS. Putative orthologs for these virulence genes were identified in all strains regardless of phenotype, and in most cases, the genes were 100 % identical at the nucleotide-level. Even in cases where variability was detected, we failed to identify any mutations that would likely result in the loss of function. Notably, Bp 576 showed deletions in one locally repetitive region amounting in 99 deleted bases in BPSS1493 (a gene associated with actin motility and just downstream from *bimA*), as compared to Bp K96243 (data not shown). Although this deletion does not disrupt the coding frame, the functional consequences of this mutation need to be empirically determined. In addition, we cannot exclude the impact of 37 *in silico* identified T3SS proteins that may contribute to the virulence of *Burkholderia* spp. [56, 57]. The genome integrity and the function of these putative factors require further investigation. Importantly, to generate a strong correlation between virulence and MNGC/actin tail formation, a larger number of the *Burkholderia* strains will need to be incorporated for *in vivo* virulence and *in vitro* phenotype studies.

Cytokines produced during the course of *Burkholderia* infection behave like a double-edged sword. Select cytokines, while important for resistance to *Burkholderia* infection, are also potential contributors to immunopathology [52]. Infection of RAW264.7 macrophages with the different *Burkholderia* spp. resulted in uniform increased secretion of IL-1β, TNF-α and IL-8. However, macrophages infected with Bm strains showed a statistically significant increased production of IL-6 and IL-10 compared to Bp strains. The induction of IL-6 cytokines was also observed in non-human primate (NHP) peripheral blood mononuclear cells (PBMCs) infected with Bm or stimulated with Bm derived LPS [58]. Further, gene expression profiling of eighty-four inflammation related genes identified 29 host genes that exhibited differential responses between the *Burkholderia* strains. Validation studies confirmed Bm mediated increased expression of *Il-1b*, *Il-10, Tnfrsf1b* and *Il-36a* host genes compared to Bp.

In addition to the virulence factors, the Bp genome encodes about 627 genes on chromosome 1 and 819 genes on chromosome 2 that are either not present or variant in Bm. A majority of these genes function in amino acid, nitrate, tagatose, allantoin and cellobiose metabolism. In addition, several genes (e.g.: Succinate-semialdehyde dehydrogenase, Glycerate kinase 1, Succinate-semialdehyde dehydrogenase, D-3-phosphoglycerate dehydrogenase, etc.) are involved in glycolysis and tricarboxylic acid (TCA) cycle [41]. Recent studies have revealed that metabolites can regulate innate immune responses [59]. For example, stimulation of macrophages by LPS, a component of the outer membrane of Gram-negative bacteria, upregulates succinate, a TCA cycle intermediate. Inhibition of prolyl hydroxylases (PHDs) activity by succinate stabilizes HIF-1α, a transcription factor that binds to the IL-1β promoter and triggers IL-1β production [59]. We hypothesize that the fundamental differences in the metabolic capabilities between Bm and Bp may affect the host metabolite profile and delineate the observed cytokine and gene expressions levels.

Bp also activates host innate immune responses through two NLRs, NLRC4 and NLRP3 [47]. Bp mediated NLRC4 activation induces pyroptosis that restricts intracellular bacterial growth whereas its engagement to NLRP3-inflammasome promotes IL-1β production that may lead to tissue damage. NLRP3 has been suggested in multiple metabolic diseases [60]. In addition to pathogen associated molecular patterns, NLRP3 can also be activated by metabolic “danger” signals such as high levels of glucose, saturated fatty acids and ceramides that are typically associated with obese or diabetic individuals. Since diabetes is a major risk factor of melioidosis, comparative analysis to characterize Bp vs. Bm mediated cytokine and gene expression changes will provide insight toward understanding of innate immunity and disease progression.

## Conclusions

These studies provide a detailed analysis, at the cellular and immunological level, the ability of a diverse range of pathogenic and avirulent *Burkholderia* strains to infect and trigger host immune responses in murine RAW264.7 macrophages. However, multiple challenges remain, as identifying gene sets or phenotypic alterations that can be used to profile the diverse *Burkholderia* strains or species and generate characteristic molecular or cellular signatures, as described in this study, still require further investigation and validation. Furthermore, a large number of the *Burkholderia* strains will need to be evaluated for statistical analysis that will help generate characteristic signature profile linking the observed cellular or molecular phenotype to the source or geographical location at either the strain or species level or the virulence.

## Methods

### Bacterial strains and macrophage culture

*Burkholderia* strains used in this investigation were obtained from the Department of Defense Unified Culture Collection (UCC) maintained at USAMRIID. Bm ATCC 23344 *ΔwcbB*, Bm ATCC 23344 *ΔwbiL* and Bm ATCC 23344 *ΔwbiL/ΔwcbB* were obtained from the laboratory of Mark Schell, University of Georgia. Detailed information about these strains can be found in Table 1 and Additional file 7: Table S4. *Burkholderia* cultures were maintained on Luria Broth (LB) plates with 1.5 % agar or on sheep blood agar (SBA) plates containing 5 % sheep blood. All Bm strains were cultured on LB containing 4 % glycerol. Agar plates were incubated at 37 °C and broth cultures were grown at 37 °C with shaking at 250 rpm. Bacterial concentrations were quantified using OD_600_ readings and diluted using a conversion factor of 5 × 10^8^ CFU/ml per unit of optical density at 600 nm [61]. All studies using viable Bp and Bm strains were performed using biosafety level three conditions.

RAW264.7 macrophage cell line (ATCC, Manassas, VA) were maintained at 37 °C with 5 % CO_2_, in DMEM (Life Technology, Carlsbad, CA) containing 10 % fetal bovine serum (FBS) (Hyclone, Logan, UT), 1 % nonessential amino acids (Sigma-Aldrich, St. Louis, MO), and 1 % glutamax (Life Technology, Carlsbad, CA).

### Colony forming unit (CFU) assay to quantify bacterial uptake and intracellular replication

RAW264.7 macrophages were seeded in 96 well (2 × 10^4^ cells/well for Bm infections) or 24 well (2.5 × 10^5^ cells/well for Bp, Bt and Bo) tissue culture plates and incubated overnight at 37 °C with 5 % CO_2_. Bacterial survival for each strains was performed using a modified Km protection assay [62]. RAW264.7 macrophages were infected by *Burkholderia strains* with a multiplicity of infection (MOI) of 10. Two hours post-infection, macrophages were washed three times with PBS and either lysed using 0.1 % (vol/vol) Triton X-100 (Sigma-Aldrich, St. Louis, MO) or incubated with pre-warmed DMEM containing 10 % FBS and 250 μg/ml of Km. At 4 and 8 h post-infection, macrophages were washed two times with PBS and lysed with 0.1 % (vol/vol) Triton X-100. Serial dilutions of the lysates were performed and plated onto SBA plates. After incubation for 48 h at 37 °C, colonies were counted and CFU/ml (CFU/ml is the number of colonies on the plate multiplied by the dilution factor and adjusted to a volume of 1 ml) was computed by normalizing to input CFUs based on colony counts.

### Immuno-fluorescence staining

RAW264.7 macrophages were infected by *Burkholderia* spp. at a MOI of 10 for ten hours. Macrophages were washed twice with PBS and fixed with formaldehyde. Antibodies AB-BURK-P-MAB3 (ABE#393, Critical Reagents Program, Frederick, MD), AB-G-BURK-M (ABE#327, Critical Reagents Program, Frederick, MD) and phalloidin-568 (Life Technology, Carlsbad, CA) were used to detect Bp, Bm and actin filaments, respectively. Images were acquired using an Opera confocal reader (model 3842-Quadruple Excitation).

Methods for quantifying bacteria and MNGCs were described previously [48, 63]. Briefly, to detect and quantify cell associated and internalized bacteria, Acapella’s Spot Detection algorithm was used. For MNGC quantitation, RAW264.7 macrophages whose nuclei are at a distance of 0–3 pixels were considered as part of a single cluster. Cellular attributes of the cell population were then imported (as sums) into the corresponding clusters and the number of nuclei per cluster attribute calculated. Clusters were then further classified into a MNGC subpopulation based on the number of nuclei present in the cluster (nuclei per cluster >3). The percentage of MNGC was calculated as (Number of MNGC objects)/(Number of Cluster objects)*100. The number of nuclei represents the total number of cells that were imaged and data acquired.

### Statistical analysis of the High Content Imaging data

While a majority of the data was approximately normal, there were a few data points that were apparent outliers. In order to obtain accurate estimates of the means and standard deviations that were not unduly influenced by these extreme data points, a robust Bayesian approach was used that fit a *t* distribution to the residual error. Using *t*-distributed error rather than normal error allows the use of the degrees of freedom parameter, *ν*, to act as a normality parameter that is low in the presence of outliers and high when the data are more normal. Let *i* index Day, *j* index Plate, and *k* index strain, then the Day effect was fit first using the following model:

$$ {y}_{ijk}\sim t\left({\mu}_i,{\sigma}_i,{v}_i\right) $$

Where *y*_*ijk*_ is the response on the *i*^th^ day, the *j*^*th*^ plate, and the *k*^*th*^ plate, *μ*_*i*_ is the mean effect for day *i*, *σ*_*i*_ is the standard deviation, and *ν*_*i*_ is the normality parameter. The priors are

$$ \begin{array}{l}{\mu}_i\sim Normal\left(\frac{\overline{y}}{1000},1000\cdot {\widehat{\sigma}}_y^2\right)\\ {}{\sigma}_i\sim Uniform\left(\frac{{\widehat{\sigma}}_y^2}{1000},1000\cdot {\widehat{\sigma}}_y^2\right)\\ {}{v}_i\sim Exponential\left(\frac{1}{29},1\right)\end{array} $$

The prior for *ν*_*i*_ is a shifted exponential with lower bound at 1. After fitting the Day effect, the median of the posterior distribution of *μ*_*i*_ is subtracted from *y*_*ijk*_ and that difference, *y*_*ijk*_^(1)^, is fit using the same methods for the Plate effect, with the median of the distribution of the average Plate effect, *μ*_*j*,_ subtracted from *y*_*ijk*_^(1)^, and the result, *y*_*ijk*_^(2)^ is used to fit the Strain effect using the same method.

The posterior distributions of the mean Strain effect are plotted in violin plots. Posterior distributions represent the probability distribution of the parameter and confidence intervals are derived using the quantiles of the posterior. Violin plots are more informative as to the actual distribution, with the thickest portions of the violin plot representing the highest probability regions for the parameter - in this case the mean of the strain effect. Taller thinner violin plots represent estimates that are more variable and less certain than shorter wider violin plots on the same scale.

### Gene transcription analysis using Real-time PCR

RAW264.7 macrophages (1 × 10^6^ cells/well) were untreated (negative control), treated with 1 μg/ml *Escherichia coli* LPS (Life Technology, Carlsbad, CA) or infected with *Burkholderia* spp. at a MOI of 10. At 4 and 8 h post-infection, RNA was isolated from RAW264.7 macrophages using Trizol® (Life Technology, Carlsbad, CA) according to the manufacturer’s protocol. Approximately 1 μg of purified RNA was subjected to genomic DNA elimination and cDNA synthesis using the RT^2^ first strand kit (Qiagen, Valencia, CA). cDNA was added to RT^2^ qPCR master mix and 25 μl was added to each well of a RT^2^ profiler mouse inflammatory chemokines and receptors plate (Qiagen, Valencia, CA). PCR amplification was performed using Applied Biosystems 7900 HT Real-time PCR instrument (Life Technology, Carlsbad, CA).

### Statistical analysis of the real-time PCR data

The raw Ct (threshold cycle) data for 84 inflammation-related genes in *Burkholderia*-infected RAW264.7 macrophages were corrected for housekeeping genes to yield a ΔCt dataset. This data was further corrected by subtracting the average values for the untreated controls for the 4 and 8 h time points (ΔΔCt). ΔΔCt values were z-scored (absolute deviation) normalized within each strain and hierarchically clustered (Manhattan distance calculation and a Wards linkage method) across gene expression (N = 84) and *Burkholderia* strains (N = 20). Selective grouping of neighboring branches within the cluster dendrogram for *Burkholderia* strains was used to determine sub-clade members that yield significant (p < =0.05) differences in gene expression when compared to other sub-clade members (this procedure is automatically implemented within the pvclust package in the R Programming Language) [64]. This method reduced the original dendrogram of 20 *Burkholderia* strains and *E. coli* LPS control into 11 sub-clades. The fidelity of these 11 sub-clades was evaluated using linear discriminant analysis (lda), neural nets (nn) and random forests (rf). The consensus amongst these methods found general agreement, with exact predictions for all but 3 of the 11 sub-clades, and a positive predictive value of greater than 0.6 for the exceptions. A separate application of stepwise linear anova regression modeling (sub-clades(1–11) ~ gene expression) yields a multiple R-squared value of 0.9706 and an adjusted R-squared value of 0.9366, corresponding to an F-statistic of 28.53 and a *p*-value of 2.811e-10. The magnitude of this *p*-value further supports the classification of *Burkholderia* strains into sub-clades (1–11). Grouping gene expressions according to sub-clades (1–11), and performing all-to-all pairwise Student’s *t*-test (number of pairwise comparisons = 11*10/2 = 55), is used to identify genes having a significantly (p < 0.05) different expression between at least one pair of sub-clades.

### Quantitation of cytokine production

RAW264.7 macrophages (1 × 10^6^ cells/well) were infected with indicated *Burkholderia* spp. at a MOI of 10. Eight hours post infection, supernatants were collected and filtered using low protein binding 0.2 μm filters. Mouse cytokines were measured using Meso-Scale Discovery Ultrasensitive pro-inflammatory 7-plex plates with an I2400 plate-reader (Meso Scale Discovery, Gaithersburg, Maryland).

### Comparative Genomics analyses of *Burkholderia* spp.

A nucleotide-level BLAST (NCBI) was used to identify putative orthologs of virulence factors within each of the available genomes. When divergence was seen between the query sequence and the subject genome, a modified version of Psi-Fi was used to identify to mutations that would likely result in the loss of gene function [65]. NCTC10229 (NC_008835-NC_008836) was used as the query strain for all *B. mallei* genomes (Bm NCTC 10247 and Bm NCTC 3709) and K96243 (NC_006350-NC_006351) was used as the query strain for all Bp genomes (Bp 576, Bp 1026b) (Additional file 5: Table S2). Bp 295, Bp DD50, Bp 776 and Bp 713 were not included in the analysis due to the lack of sequence availability.

### Transmission Electron Microscopy (TEM)

RAW264.7 macrophages (2 × 10^6^ cells/well) were infected with Bp K96243 and Bp 776 at MOI 10. After 2 h incubation, cells were washed 2x and media containing 250ug/ml KAN was added to kill extracellular bacteria. Cells were scraped with a cell lifter, washed, pelleted, and fixed at room temperature for 1 h in TEM primary fixatives (2.5 % formaldehyde, 2.5 % glutaraldehyde in 0.1 M sodium cacodylate pH 7.4 buffer) at each of the following time points: 2, 4, 6, and 8 h. Cells were then washed three times in 0.1 M sodium cacodylate buffer for 10 min each, the primary fixed cells were then incubated with 1 % Osmium Tetroxide in 0.1 M Sodium Cacodylate for 1 h. After washing with distilled water three times for 10 min each, fixed cells were stained in 1 % uranyl acetate for 1 h and dehydrated in ethanol series of 22, 50, 75 and 95 % successively for 10 min each. The cells were dehydrated three times for 10 min each in 100 % ethanol and then two times for 10 min each in propylene oxide. Cells were infiltrated in well mixed 50 % propylene oxide, 50 % Epon812 (Electron Microscopy Sciences, RT14120) for 1 h with agitation at room temperature followed by 100 % Epon 812 3 times for 1 h each with agitation. After which the samples were placed in an oven and allowed to polymerize at 60 °C for 24 h. Thin sections (approximately 80 nm) were collected and pre-stained with 1 % uranyl acetate and Sato lead before examination in a JEOL 1011 transmission electron microscope at 80kv and digital images were acquired using AMT camera system.

### Ethics statement

As no human or animal subjects were used for this work consent and ethical approval was not required.

### Availability of supporting data

## Additional files

Additional file 1: Figure S1. Bp K96243 and Bp 776 escape from membrane bound vesicles. (A) RAW264.7 macrophages were infected by either Bp 776 or Bp K96243 at indicated time points. Samples were prepared and subjected to TEM as described in the methods section. The scale bar represents 0.5 μm. Arrows indicate bacteria. (B) A total number of 25 cells were examined to determine the number of Bp that reside either inside or outside of membrane bound vesicles. (TIFF 3607 kb) Additional file 2: Figure S2. Statistical analysis of the cellular attributes of the MNGC formation. Paired Student’s *t*-test was performed to evaluate the probability that the mean value of the strain listed in the column is greater than the strain listed in the row for the feature % MNGC formation. Data for the 10 h post infection is shown. (TIFF 160 kb) Additional file 3: Figure S3. Statistical analysis of cytokine production in RAW264.7 macrophages infected with *Burkholderia* spp. Paired Student’s *t*-test was performed to evaluate the statistical significance of differential cytokine productions after infecting RAW264.7 macrophages with indicated *Burkholderia* spp. (TIFF 571 kb) Additional file 4: Table S1. Hierarchical clustering of the inflammation related gene expression data. This is the underlying data for Fig. 6. The raw Ct data for 84 inflammation-related genes in *Burkholderia*-infected RAW264.7 macrophages were corrected for housekeeping genes to yield a ΔCt dataset. This data was further corrected by subtracting the average values for the untreated controls for the 4 and 8 h time points (ΔΔCt). ΔΔCt values were z-score (absolute deviation) normalized within each strain. Explanation of the column headings: column B to column AQ represents the name of the *Burkholderia strain* and the time point at which the expression of genes were assayed. (XLSX 50 kb) Additional file 5: Table S2. Pairwise comparison of p-values between *Burkholderia* strain for each gene at 4 and 8 h. Column headings specify gene, strain 1, strain 2, p-value, t-statistic and exposure time. Left and right columns correspond to the 4 and 8 h exposure times, respectively. T-statistic is based on comparisons of strain 1 to strain 2. *P*-values less than 0.2 are listed. (XLSX 16 kb) Additional file 6: Table S3. A list of loci examined in comparative genomic study. (XLSX 17 kb) Additional file 7: Table S4. UCC nomenclature for the different *Burkholderia* spp. Explanation of the column headings: Burkholderia Species: names of the species used in this study; Strain names: names of the strains; UCC nomenclature: the corresponding nomenclature used within USAMRIID. (XLSX 10 kb)

## Abbreviations

Bm: *Burkholderia mallei*
Bo: *Burkholderia oklahomensis*
Bp: *Burkholderia pseudomallei*
Bt: *Burkholderia thailandensis*
CFU: Colony forming unit
CHBP: Cif homolog in Bp
HCI: High-content imaging
IFN: Interferon
LPS: lipopolysaccharide
MNGC: Multinucleated giant cell
NHP: Non-human primate
NLR: NOD-like receptor
PBMC: Peripheral blood mononuclear cell
PHD: Prolyl hydroxylase
PRR: Pathogen recognition receptor
T3SS: Type III secretion system
T6SS: Type VI secretion system
TCA: Tricarboxylic acid
TEM: Transmission electron microscopy
TLR: Toll-like receptor
