# Supplementary figures and images for: Characterization of the murine macrophage response to infection with virulent and avirulent Burkholderia species
Source: BMC Microbiol. 2015 Nov 6;15:259. doi: 10.1186/s12866-015-0593-3 (PMC4636792; doi:10.1186/s12866-015-0593-3)

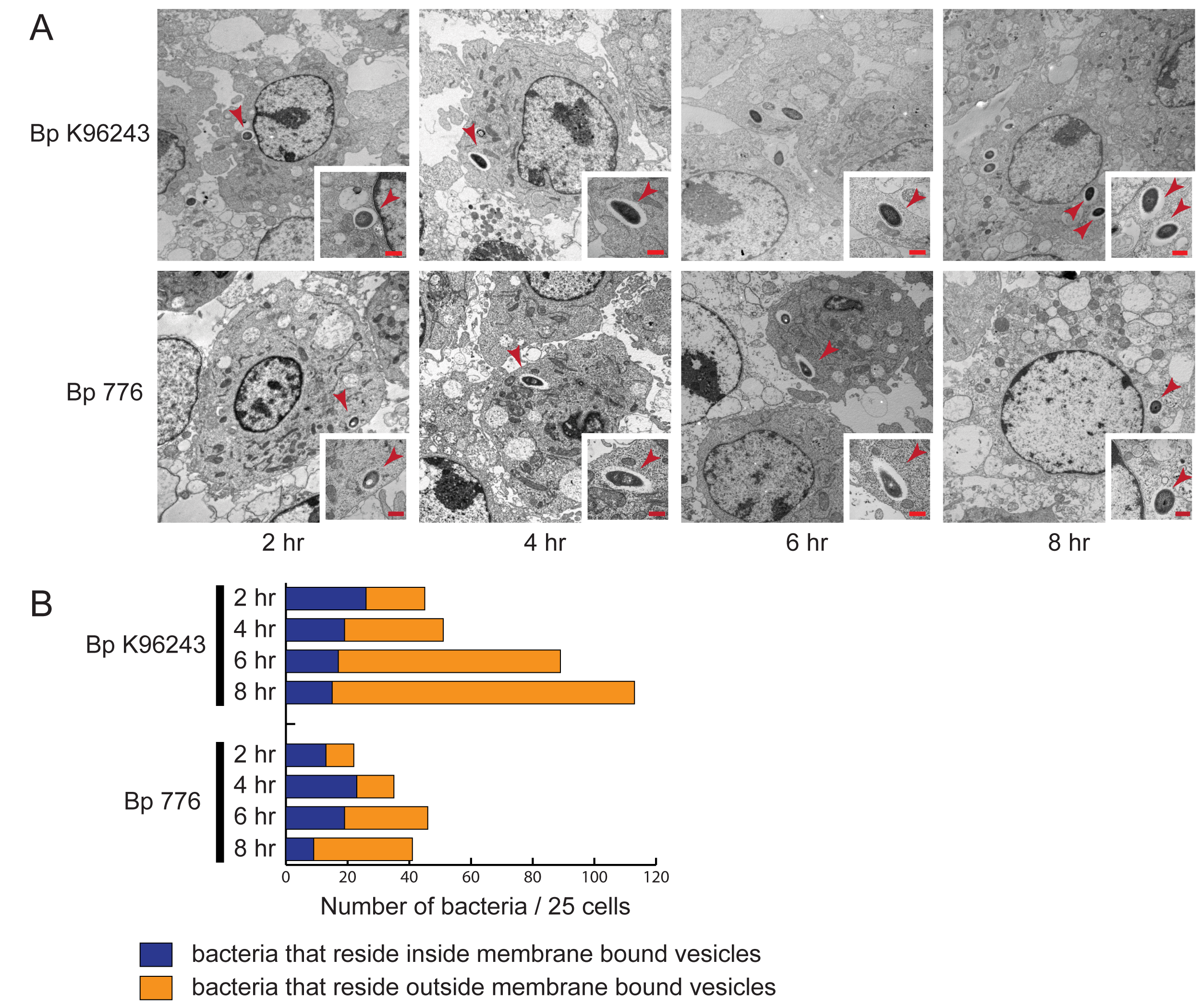

Supplement: Additional file 1: Figure S1. — Bp K96243 and Bp 776 escape from membrane bound vesicles. (A) RAW264.7 macrophages were infected by either Bp 776 or Bp K96243 at indicated time points. Samples were prepared and subjected to TEM as described in the methods section. The scale bar represents 0.5 μm. Arrows indicate bacteria. (B) A total number of 25 cells were examined to determine the number of Bp that reside either inside or outside of membrane bound vesicles. (TIFF 3607 kb) [file 12866_2015_593_MOESM1_ESM.tif]

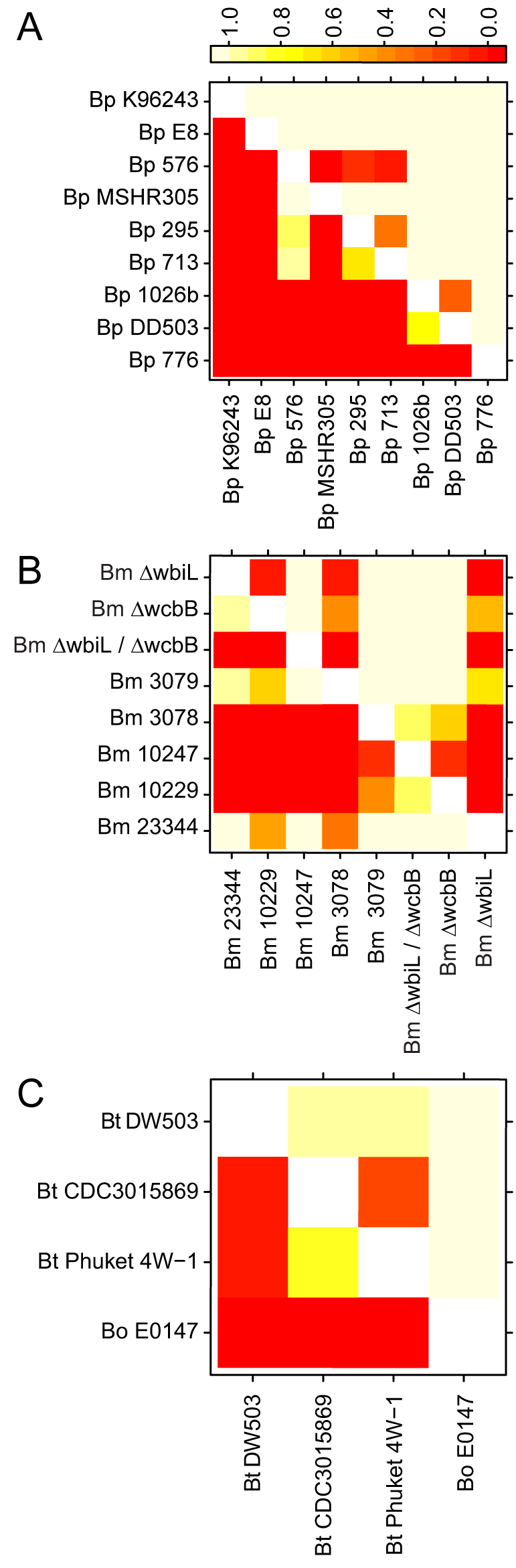

Supplement: Additional file 2: Figure S2. — Statistical analysis of the cellular attributes of the MNGC formation. Paired Student’s t-test was performed to evaluate the probability that the mean value of the strain listed in the column is greater than the strain listed in the row for the feature % MNGC formation. Data for the 10 h post infection is shown. (TIFF 160 kb) [file 12866_2015_593_MOESM2_ESM.tif]

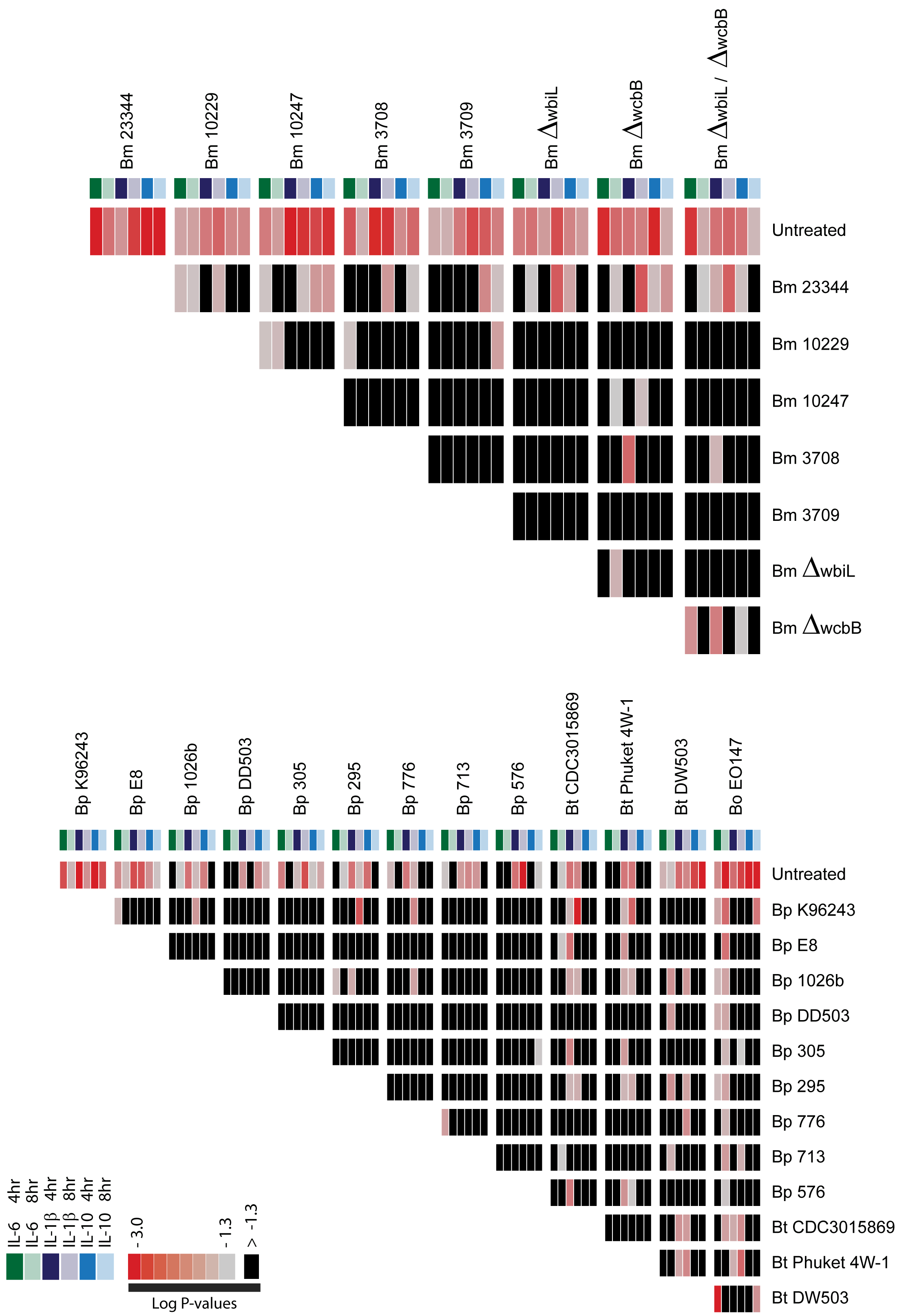

Supplement: Additional file 3: Figure S3. — Statistical analysis of cytokine production in RAW264.7 macrophages infected with Burkholderia spp. Paired Student’s t-test was performed to evaluate the statistical significance of differential cytokine productions after infecting RAW264.7 macrophages with indicated Burkholderia spp. (TIFF 571 kb) [file 12866_2015_593_MOESM3_ESM.tif]
